# On the Strong Binding Affinity of Gold-Graphene Heterostructures with Heavy Metal Ions in Water: A Theoretical and Experimental Investigation

**DOI:** 10.1021/acs.langmuir.4c02568

**Published:** 2024-09-13

**Authors:** Tommaso Del Rosso, Ivan Shtepliuk, Quaid Zaman, Luis Gonzalo Baldeón Huanqui, Tahir, Fernando Lazaro Freire, Andre Nascimento Barbosa, Marcelo Eduardo Huguenin Maia da Costa, Ricardo Q. Aucélio, Jarol Ramon Miranda Andrades, Cesar D. Mendoza, Rajwali Khan, Giancarlo Margheri

**Affiliations:** † Department of Physics, Pontifícia Universidade Católica do Rio de Janeiro, Rua Marques de São Vicente, 22451-900, Rio de Janeiro, Brazil; ‡ Semiconductor Materials Division, Department of Physics, Chemistry and Biology - IFM, Linköping University, S-58183 Linköping, Sweden; § Department of Physics, Main Sowari Bazzar, University of Buner, 17290 Buner, Pakistan; ∥ Department of Chemistry, Pontifícia Universidade Católica do Rio de Janeiro, Rua Marques de São Vicente, 22451-900 Rio de Janeiro, Brazil; ⊥ Departamento de Engenharia Elétrica, Universidade do Estado do Rio de Janeiro, UERJ, Rua São Francisco Xavier 524, Maracanã, Rio de Janeiro 20550-900, RJ Brazil; # National Water and Energy Center, United Arab Emirates University, P.O Box 17551, Sheik Khalifa Bin Zayed Street 1, Al-Ain, United Arab Emirates; @ Istituto dei Sistemi Complessi Sezione di Sesto Fiorentino (I.S.C - CNR), Via Madonna del Piano 10, 50019 Sesto Fiorentino, Italy

## Abstract

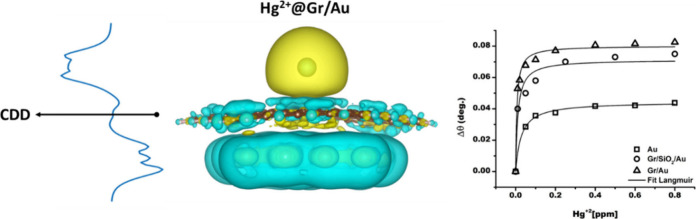

Minimum energy configurations in 2D material-based heterostructures can enable interactions with external chemical species that are not observable for their monolithic counterparts. Density functional theory (DFT) calculations reveal that the binding energy of divalent toxic metal ions of Cd, Pb, and Hg on graphene-gold heterointerfaces is negative, in contrast to the positive value associated with free-standing graphene. The theoretical predictions are confirmed experimentally by Surface Plasmon Resonance (SPR) spectroscopy, where a strong binding affinity is measured for all the heavy metal ions in water. The results indicate the formation of a film of heavy metal ions on the graphene-gold (Gr/Au) heterointerfaces, where the adsorption of the ions follows a Langmuir isotherm model. The highest thermodynamic affinity constant *K* = 3.1 × 10^7^ L mol^–1^ is observed for Hg^2+^@Gr/Au heterostructures, compared to 1.1 × 10^7^ L mol^–1^ and 8.5 × 10^6^ L mol^–1^ for Pb^2+^@Gr/Au and Cd^2+^@Gr/Au, respectively. In the case of Hg^2+^ ions, it was observed a sensitivity of about 0.01°/ppb and a detection limit of 0.7 ppb (∼3 nmol L^–1^). The combined X-ray photoelectron spectroscopy (XPS) and SPR analysis suggests a permanent interaction of all of the HMIs with the Gr/Au heterointerfaces. The correlation between the theoretical and experimental results indicates that the electron transfer from the graphene-gold heterostructures to the heavy metal ions is the key for correct interpretation of the enhanced sensitivity of the SPR sensors in water.

## Introduction

Surface plasmon polaritons (SPPs) are p-polarized electromagnetic waves propagating along metal-dielectric interfaces, supported by the collective oscillations of free electrons in metals.^1^ The SPPs are characterized by an evanescent wave extending for hundreds of nanometers in the external medium, which can be gaseous or liquid.^1^ The study of the perturbation of the evanescent field upon changes of the refractive index of the external medium is at the basis of the SPR and localized SPR (LSPR) spectroscopies and has been widely used in the literature for the development of biosensors, environmental and radiation sensors, and characterization of nanomaterials and thin films, including 2D structures.^2−13^

In particular, there are several studies on graphene-supported plasmonics, where the presence of graphene (Gr) is used to improve both the stability and the sensitivity of the SPR sensing platforms.^9,14−16^ However, pure graphene is known for its strong chemical inertness, so that most of the literature is dedicated to the interaction between analytes and carbon nanostructures with amino or carboxyl functional groups, such as graphene oxides (GO) and reduced graphene oxides (rGO).^17−20^ A common approach is the synthesis of graphene-based heterostructures with noble metals or metal oxides to improve the stability, reproducibility, and resolution of the devices in sensing applications. This approach is based on the changes in morphology, surface chemistry, and/or conductivity of the heterostructures compared to isolated GO or rGO.^21−26^

Particularly interesting for its originality is the theoretical report from Ivan Shtepliuk et al.^26^ Herein, using a supercell approach, it is shown that charged toxic metal ions (here referred also as heavy metal ions or “HMIs” following a more general but rather nonprecise term) behave as electron acceptors on infinite pure free-standing graphene, with binding energies typical for chemisorption (>0.5 eV). These theoretical results suggest the possibility of developing new sensing strategies based on the modulation of the binding energy of selected analytes on pure graphene, without the use of GO or rGO.

The case of HMIs in water is of great interest due to the toxicity to humans and to the environment, as they are used in several industrial activities.^27,28^ In the past decade, great efforts have been dedicated to establishing portable and cheap technologies based on different nanomaterials and devices, to develop an alternative to classical analytical methods based on expensive techniques such as inductively coupled plasma mass spectrometry (ICP-MS).^29^ The efforts are directed toward the development of HMIs sensors with a limit of detection (LOD) at the ppb level, considering that, for example, 2.0 ppb of mercury ions (Hg^2+^) is considered as the maximum concentration allowed for drinking water by the United States Environmental Protection Agency (EPA).^30^ For this aim, different physical approaches can be found in literature, based, for example, on photoluminescence spectroscopy,^31−33^ electrical measurements,^25−34^ or SPR spectroscopy.^20,35,36^ In this context, new sensing approaches can be derived from an understanding of the chemical physics underlying the interaction of the HMIs with the surfaces of the sensors.

In this paper, we propose a different strategy to enhance the adsorption capacity of HMIs on graphene-based thin film devices based on the study of the binding energy of the HMIs on graphene-gold (Gr/Au) heterostructures supporting surface plasmon polaritons. To investigate the feasibility of this proposal, we start the results and discussion section with density-functional theory (DFT) calculations applied to the interaction between HMIs and free-standing graphene or Gr/Au heterostructures, which show how the presence of the noble metal substrate can modulate the binding energy and charge transfer with Hg^2+^, Pb^2+^, and Cd^2+^. In the light of the theoretical results, an experimental verification by measuring the binding affinity of the HMIs by SPR spectroscopy, where a Langmuir isotherm model is applied for the adsorption of the ions^35^ on the different heterostructures, was proposed. Finally, the correlation between the DFT results and the experimental performance of the SPR sensors was discussed.

## Materials and Methods

### Materials

SiO_2_ and gold pellets were purchased from Kurt J. Lesker Company with a purity better than 99%. Ethanol, acetone, trichloroethylene, tetrahydrofuran (THF), iron(III) chloride, and 3-mercaptopropyltrimethoxysilane (MPTS) were purchased from Sigma-Aldrich. Polyurethane (PU) pellets were purchased from BASF, and copper foils of 25 μm thickness, used for graphene synthesis, were purchased from Alfa Aesar with 99.8% purity. Deionized water was obtained from a Milli-Q purification system, Millipore, USA.

### Fabrication of the Gr/Au Heterostructures

The protocol for the fabrication of the Gr/Au and Gr/SiO_2_/Au heterostructures supporting the propagation of the plasma waves is reported in detail in refs (9,16). Briefly, SF4 glass substrates were ultrasonically cleaned and treated with a plasma cleaner to create surface hydroxyl groups (OH) useful for the subsequent deposition of a self-assembled monolayer (SAM) of MPTS. Thin films of gold (Au) with a nominal thickness of 50 nm were deposited over the SAM using a Univex 450 electron beam deposition system (p = 3 × 10^–6^ torr, deposition rate 0.5 Å s^–1^), which was also used for the deposition of a 30 nm thick SiO_2_ layer over the gold. Prior to SiO_2_ deposition, a silica-like surface was formed by self-assembly of MPTS on the gold thin film, followed by a hydrolysis and condensation process.^8^

Pure graphene (Gr) was produced by low-pressure chemical vapor deposition, using copper (Cu) foil as the substrate and methane as the precursor, with a 6:1 H_2_/CH_4_ gas flow ratio.^37^ For graphene transfer, a 1 wt % polyurethane-tetrahydrofuran (PU-THF) solution was first spin-coated onto the Cu-Gr bilayer. Subsequently, etching of copper was performed using an iron(III) chloride solution, and the obtained PU-Gr film was transferred to the gold thin films. The PU layer was finally removed by a THF bath for 4 h at 55 °C, and the resulting Gr/Au heterostructure was dried under a nitrogen gas flow.

### SPR Spectroscopy

**Figure 1:**
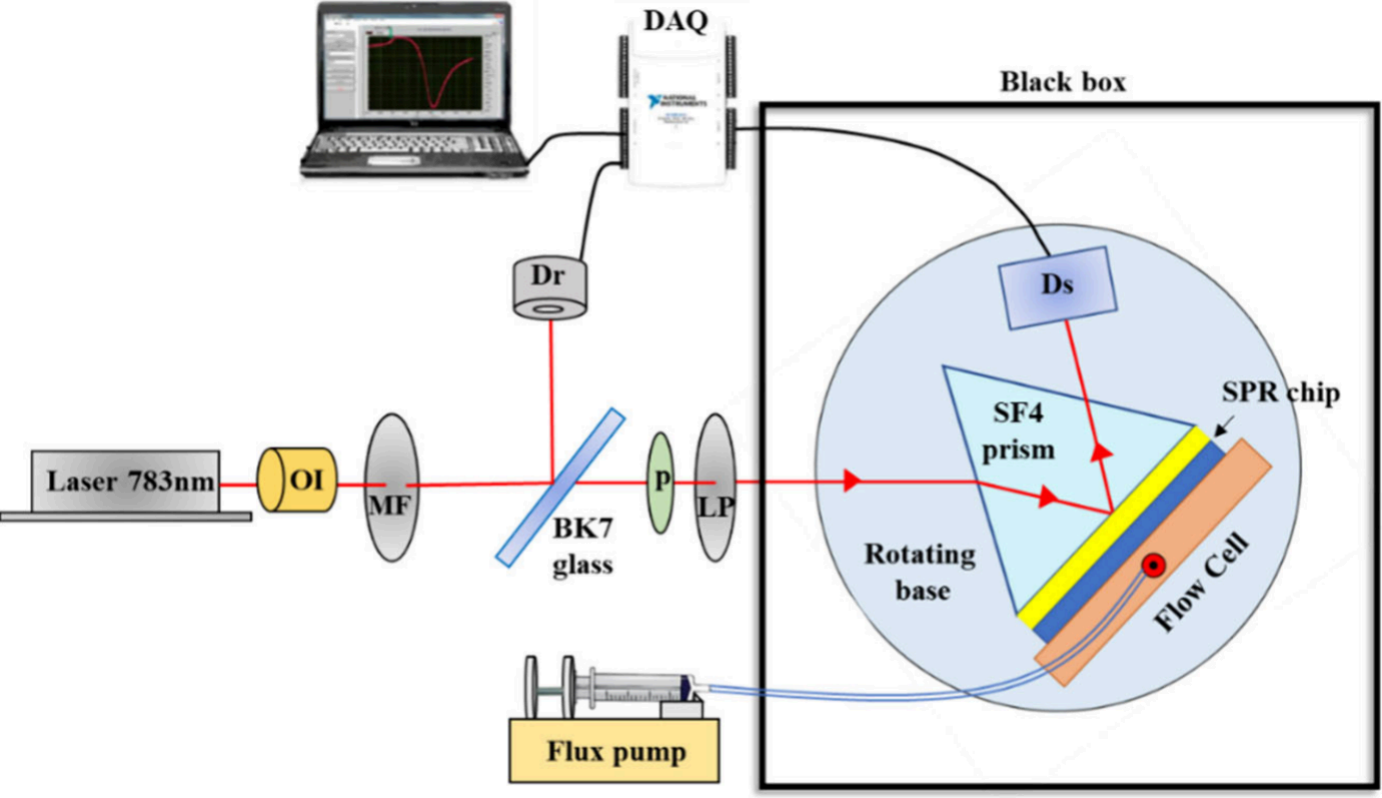
Scheme of the SPR spectrometer. OI – optical isolator; MF – metallic filter; p – pinhole; LP – linear polarizer; Dr – reference detector; Ds – signal detector; DAQ – data acquisition board.

The experimental setup used for the SPR spectroscopy in the Kretschmann configuration with angular modulation^1^ is shown in Figure 1. A diode laser (Ondax, model LM-783-PLR-75-1, USA) with an emission wavelength of 783 nm was used to excite the SPPs. The laser first passes through an optical isolator and a metal attenuator and then impinges on a BK7 glass, which reflects about 3% of the light power and transmits the remaining 97%. The reflected light reaches a reference photodetector (ThorLabs, model DET36A, USA), which measures a value proportional to the input power. The electronic signal from the reference detector is sent to a National Instruments USB-611 data acquisition board (DAQ). The transmitted light first passes through a pinhole used to align the optical system and after through a linear polarizer in TM configuration. The TM polarized light enters a black box containing a remote-controlled rotary platform from Sigma-Koki (Japan), with an angular resolution of 0.0025°. The rotary platform supports a SF4 coupling prism, the SPR sensor coupled to a 900 μL flow cell in poly(ether–ketone) (PEEK), and a signal photodetector to measure the power of the light reflected by the device (Thorlabs, model S170C, USA).

A code in LabVIEW 2017 language is used to run the angular scan of the reflected power, and the SPR spectra were analyzed by the free software Winspall 3.02^38^ used to retrieve the values of the complex dielectric constant ε = (ε_1_ + i ε_2_) and thickness of the different layers of the SPR heterostructures. In particular, for the Gr/Au samples, four layers were considered: SF4 as the first semi-infinite medium, gold as the second (finite) layer, graphene as the third (finite) layer, and water as the semi-infinite fourth medium. The SAM of MPTS is not considered in the simulations due to its transparency and its monolayer nature. In the case of Gr/SiO_2_/Au heterostructures, we considered five layers, with an additional finite layer of SiO_2_ between the graphene and the gold thin film.

### Optical Sensing of HMIs

The first experimental step consisted of the stabilization of the SPR heterostructures, obtained by immersion of the samples in deionized water along 24 h, as reported in detail in ref (9). After the stabilization, the SPR curve of the sample in deionized water was measured to obtain the reference resonance angle. Subsequently, the sensing performance of the SPR devices were investigated by the injection into the flow cell of standards of different divalent HMIs (Pb^2+^, Cd^2+^, Hg^2+^) in water, and measuring the corresponding variation in the resonance angle Δθ.

Lead nitrate (Pb^2+^), mercury chloride (Hg^2+^), and cadmium chloride (Cd^2+^) standard stock solutions were prepared at 4.0 mmol L^–1^ by dissolving appropriate amounts of the salts in deionized water acidified with HNO_3_, to obtain a final volume of 10.0 mL. From the stock solutions, 5 ppm solutions were prepared by direct dilution in deionized water, preserving the acidity of the final medium (pH ∼ 3.0). Aliquots of the 5 ppm solutions were hence injected in the flow cell filled with deionized water so that the final concentration of the HMI varied from 10 to 800 ppb. The pH of the introduced sample solutions was about 4 during the injection.

To assess the influence of the structure of the SPR device on the sensing performance, it was considered as fundamental parameters the sensitivity of the analytical response (*S*_C_) and the limit of detection (LOD), defined as^39,40^
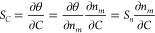
1
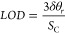
2

In eq 1, θ is the SPR resonance angle, *n*_m_ is the refractive index of the liquid medium in contact with the surface of the SPR sensor, and *C* is the concentration of the analytes. *S*_n_ represents the classical sensitivity to variation in the external refractive index expressed in °/RIU (RIU: Refractive Index Units), which was determined experimentally by measuring the angular SPR shift after the introduction of a glycerol aqueous solution with a refractive index of 1.35.^9^ In eq 2, the parameter 3δθ_r_ is the angular resolution of the system, calculated from the standard deviation of the noise of the reflected power measured by the signal detector.^3^ In this set up, 3δθ_r_ = 6.9 × 10^–3^ ° was obtained.

As reported in^20,35^ for the sensing of HMIs by SPR spectroscopy with gold thin films, it was assumed that the following Langmuir isotherm models the adsorption process on the sensor surface:
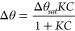
3

In eq 3, Δθ_*sat*_ is the maximum angular shift of the SPR sensor at the saturation of the binding sites, *C* is the concentration of the analytes, and *K* is the thermodynamic affinity constant. By the use of this model, it is supposed that the angular shift Δθ is proportional to the number of metal ions adsorbed on the external surface of the sensors and that the binding energy does not depend critically on the surface density of the ions.

### Raman and XPS Analysis

The quality of the graphene on copper foils and gold thin films was evaluated by Raman spectroscopy, using a micro-Raman spectrometer (NT-MDT, model NTEGRA SPECTRA, Netherlands), equipped with a 600 lines/mm diffraction grating and 520 mm focal distance, an excitation wavelength of 473 nm, and a power of less than 0.2 mW. Under the measurement conditions, the spectral resolution was 4 cm^–1^.

XPS measurements were made using a VG Thermo system with an Alpha110 hemispherical analyzer and a nonmonochromatic Al X-ray gun. The surface ejection angle adopted was normal, and the data were analyzed using CASAXPS software.^41^ Backgrounds were removed using the Shiley method, and peaks were fitted using a Voight curve.

### DFT Calculations

**Figure 2:**
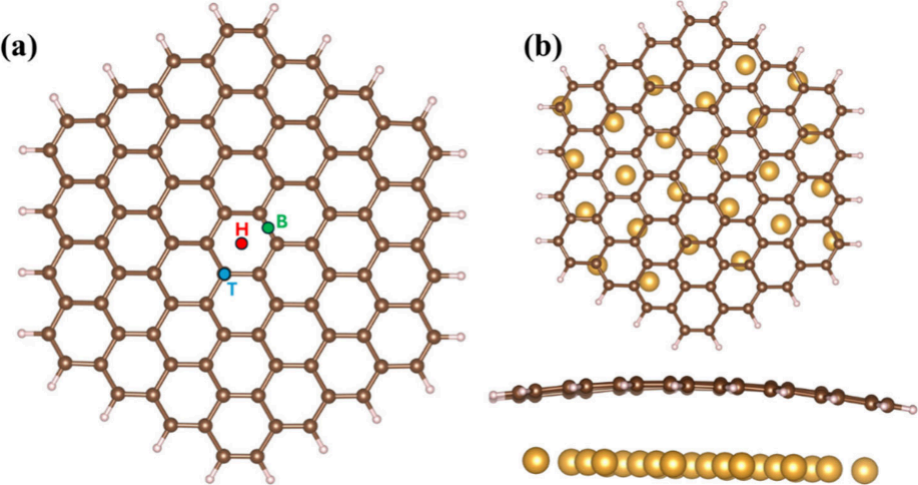
Optimized structures of (a) free-standing graphene and (b) graphene/Au(111) substrates prior to HMI adsorption, where carbon atoms are represented by brown balls, hydrogen atoms by whitish balls, and gold atoms by yellow balls.

All Density Functional Theory (DFT) calculations were performed by using Gaussian 16 Rev. B.01 package at the PBE1PBE level of theory^42^ with the Generalized Effective Core Potential (GENECP). The 6-31g(d,p) basis set was chosen for carbon and hydrogen atoms,^43^ while the SDD (Stuttgart/Dresden effective core potentials with the Dunning/Huzinaga double-ζ basis sets) basis set was selected for heavier atoms like gold, cadmium, mercury, and lead.^44^ The calculations included the empirical dispersion correction as proposed by Grimme (D3)^45^ and employed the Polarizable Continuum Model (PCM) with the SCRF = (Solvent = Water) method.^46^ The geometry optimization calculations were conducted with an SCF (self-consistent field) convergence criterion set to 10^–8^. Two possible configurations related to the adsorption of HMIs onto sensing layer surfaces were considered: HMI@Gr and HMI@Gr/Au(111). A widely adopted cluster model comprising 96 carbon atoms with hydrogen atom passivation was employed to represent graphene.^47−50^ This model is commonly chosen for studying HMI-related local adsorption phenomena on graphene.^51,52^ It serves as a reasonable and effective approach to explore these aspects of graphene’s behavior. For the design of the Gr/Au(111) structure, a single graphene layer (cluster model) containing 96 carbon atoms was placed onto a gold monolayer (with fixed Cartesian coordinates), which serves as the surface layer of the Au(111) crystal and consists of 24 gold atoms. A single atomic layer of gold was employed to represent the bulk structure, balancing the computational efficiency with physical accuracy. This approach is justified because the phenomena under investigation—heavy metal adsorption—are predominantly surface-mediated processes. The topmost atomic layer of gold plays the most crucial role in these interactions, while the influence of deeper layers is minimal. The corresponding optimized structures are demonstrated in Figure 2. Based on the nomenclature provided in ref (53), the HMIs can be positioned in three different locations on the hexagonal cell of a single graphene layer: at the top (T), bridge (B), and hollow (H) sites. The binding energy (*E*_b_) for HMI species was obtained as indicated in eq 4.
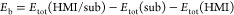
4where *E*_tot_(HMI/sub) is the total energy of substrate (Gr, Gr/Au(111)) with adsorbed HMI, *E*_tot_(sub) is the total energy of the isolated substrate in relaxed geometry, and *E*_tot_(HMI) is the total energy of an isolated HMI. With this definition, a negative binding energy denotes an energetically favorable HMI adsorption. In contrast, a positive value of binding energy may suggest a repulsive nature of the interaction between substrate and HMI species.

To get deep insights into charge distribution within Gr or Gr/Au(111) structures interacting with HMIs, the charge population analysis was carried out within two different schemes, Mulliken^54^ and Hirshfeld.^55^ Moreover, the 1D and 3D charge density difference was simulated (CDD). The 3D CDD is given by
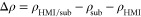
5where ρ_HMI/sub_ is the electronic density of the interacting HMI-substrate system and ρ_sub_ and ρ_HMI_ are, respectively, electron densities of the isolated substrate and HMI atom. To obtain the 1D planar (*xy*)-integrated CDD from the 3D data, the charge density difference was integrated along the *z*-direction. To gain deeper insights into the interaction between HMIs and substrates, noncovalent interaction (NCI) analysis^56^ was performed using the Multiwfn program^57^ and VMD^58^ to analyze the nature of the interactions.

## Results and Discussion

### Binding Trends and Interfacial Charge Transfer

**Figure 3:**
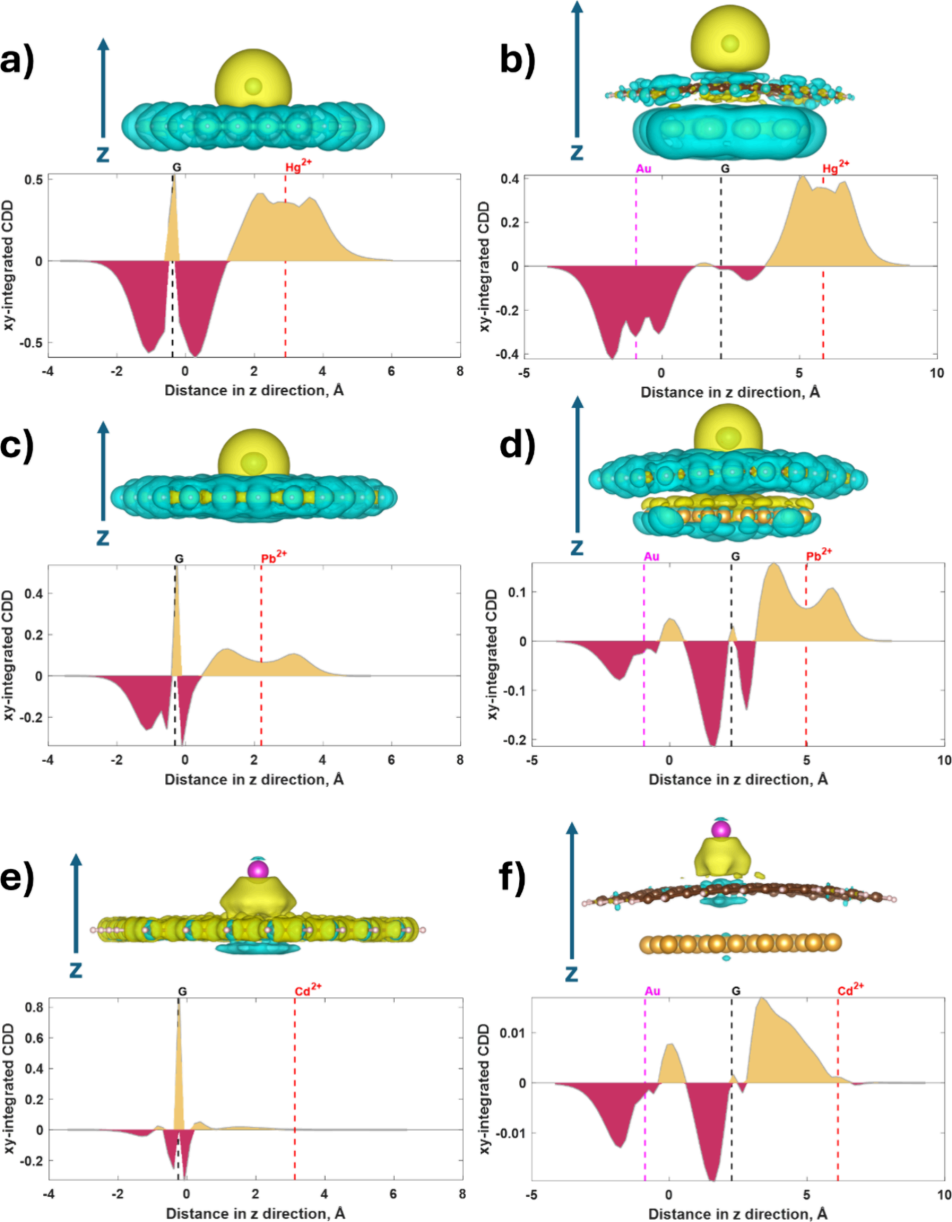
Charge density difference (CDD) associated with the following structures: a) Hg^2+^@Gr; b) Hg^2+^@Gr/Au; c) Pb^2+^@Gr; d) Pb^2+^@Gr/Au; e) Cd^2+^@Gr; f) Cd^2+^@Gr/Au. Upper panels: tridimensional CDD with the isosurface level set at 0.0001. Yellow and cyan correspond to positive and negative Δρ, where ρ is the density of the electrons. Lower panels: *xy*-integrated CDD. The direction of the *z* axes is indicated in each panel. The dashed vertical lines correspond to the location (determined as the mean *z* coordinate) of the gold layer, graphene layer, and HMI, respectively. Positive (dark-yellow) and negative (wine-colored) values of the *xy*-integrated CDD represent charge accumulation and charge depletion regions, respectively.

Values for binding energies (*E*_b_), binding sites, binding heights, and postadsorption charges on divalent cations of heavy metals (with initial charge 2+) following their interaction with graphene and graphene/Au(111) substrates are presented in Table 1. The gold layer significantly influences the adsorption characteristics of HMIs on graphene substrates. Notably, adsorption energies for Hg^2+^, Pb^2+^, and Cd^2+^ on Gr/Au(111) are predominantly negative, indicating stronger binding compared to their adsorption on free-standing graphene. This enhancement suggests that Au(111) alters the electronic environment, favoring HMI adsorption at specific sites (e.g., hollow and top sites). It is noteworthy that in the presence of a gold layer, Hg^2+^ ion demonstrates a higher binding energy compared to Pb^2+^ and Cd^2+^, despite having a greater binding height than Pb^2+^. This is because the mercury cation has a stronger chemical affinity to gold present in the graphene (binding order: Hg^2+^ > Pb^2+^ > Cd^2+^). This is further supported by the results of charge population analysis, which indicate that Hg^2+^ ions exhibit a stronger propensity to accept electrons from graphene and graphene/Au(111) substrates compared to Pb^2+^ and Cd^2+^ ions. Both Mulliken and Hirshfeld analyses yield consistent results: the postadsorption charge on the initial divalent mercury cation approaches zero, confirming its clear electron-accepting behavior. While the binding energies and charge population analyses provide valuable insights, they do not offer a complete picture of the electronic interactions between the heavy metal ions and the graphene or graphene/Au(111) substrate. CDD analysis can reveal the spatial distribution of electron transfer, giving a more detailed view of how the substrate’s electronic structure is altered upon adsorption. The 1D and 3D charge density difference plots originating from the interaction of the HMIs with free-standing graphene and Gr/Au heterointerfaces are shown in Figure 3.

**Table 1:** Parameters Describing the Binding of the HMIs on the Different Substrates

				Charge on HMI [*e*]	
Heterostructure	E_b_ [eV]	Binding site	Binding height [Å]	Mulliken	Hirshfeld
**Hg**^**2+**^**@Gr**	1.381	Hollow	3.28	0.0116	0.0717
**Hg**^**2+**^**@Gr/Au(111)**	–0.874	Hollow	3.71	–0.0393	0.0659
**Pb**^**2+**^**@Gr**	0.553	Hollow	2.50	1.407	1.1560
**Pb**^**2+**^**@Gr/Au(111)**	–0.853	Hollow	2.71	1.328	1.1493
**Cd**^**2+**^**@Gr**	0.284	Hollow	3.38	1.989	1.906
**Cd**^**2+**^**@Gr/Au(111)**	–0.233	Top	3.85	1.935	1.924

In the case of mercuric ion on graphene, analysis of the 3D CDD reveals the following pattern (Figure 3a): the mercuric ion is surrounded by a broad yellow area (indicating positive Δρ, where ρ is the electron density), while graphene is surrounded by a cyan-colored region (indicating negative Δρ). This distribution suggests net electron transfer from graphene to the mercuric ion, consistent with the electron-accepting nature of Hg^2+^. For the same case, analysis of the 1D CDD (*xy*-integrated CDD) shows a wide and intense band extending from 1.18 to 6.02 Å along the *z*-axis (positive Δρ), centered at the position (*z*-coordinate) of the mercuric ion. This extended accumulation region indicates a significant electron density perturbation around the mercury ion, suggesting a strong interaction with the graphene substrate. Additionally, a narrow positive peak corresponds to the position of graphene surrounded by relatively wide regions of negative Δρ. This pattern implies local polarization of the graphene sheet, with electron density being drawn toward the adsorbed mercuric ion. Since positive and negative values of CDD represent charge accumulation and depletion regions, respectively, it is evident that the charge primarily accumulates around the mercuric ion. The extended nature of this accumulation region suggests that the interaction between Hg^2+^ and graphene is not purely electrostatic but involves significant orbital overlap and hybridization. Interestingly, in the presence of a gold layer (Figure 3b), significant charge accumulation still occurs around the mercuric ion, while the previously mentioned narrow positive peak disappears. Instead, the charge depletion region is mainly located around the gold layer. This redistribution indicates that the gold substrate plays a crucial role in modifying the electronic interaction between Hg^2+^ and graphene, potentially explaining the enhanced binding energy observed in the Hg^2+^@Gr/Au(111) system.

Analyzing the case of lead ion on free-standing graphene (Figure 3c), a similar pattern was observed, albeit with less intense charge accumulation (indicating lower charge transfer). This reduced charge transfer correlates with the lower binding energy of Pb^2+^ compared to Hg^2+^, suggesting a weaker interaction with the graphene surface. However, the adsorption of Pb^2+^ on Gr/Au(111) (Figure 3d) leads to greater charge depletion around graphene compared to around the gold layer, as observed in Hg^2+^@Gr/Au(111). This difference in charge redistribution between Hg^2+^ and Pb^2+^ on Gr/Au(111) could explain their different adsorption behaviors and might be exploited for selective adsorption in practical applications.

In contrast, significant charge accumulation around the cadmium ion adsorbed on both substrates was not observed (Figure 3e and Figure 3f), indicating much weaker charge transfer. This weak interaction aligns with the lower binding energy of Cd^2+^. It suggests that its adsorption might be more easily reversible, which could be advantageous in certain environmental remediation scenarios, where regeneration of the adsorbent is desired. Nevertheless, the presence of Cd^2+^ adsorbed on free-standing graphene causes localized charge redistribution in graphene (see the narrow intense positive peak in Figure 3e, centered at the position of graphene and surrounded by two narrow charge depletion regions). Despite the overall weak interaction, this local polarization might still provide sufficient binding for Cd^2+^ sensing applications, albeit with an efficiency lower than that for Hg^2+^ and Pb^2+^. Interestingly, in the presence of gold, the charge transfer is even further reduced (Figure 3f), correlating with the results of the charge population analysis. This observation underscores the complex role of the gold substrate in modulating the adsorption characteristics of different toxic metal ions, which could be leveraged for designing more selective and efficient adsorption materials.

The observed extended charge accumulation regions, particularly for mercury and lead, have significant practical implications. They suggest that the adsorbed ions may retain some of their reactivity due to the diffuse nature of the charge transfer. This could be beneficial in sensing applications or in scenarios where subsequent reactions of the adsorbed species are desired. The differences in the extent and distribution of these regions among different ions provide a basis for developing selective sensors or adsorbents tailored to specific metal contaminants.

To complement the CDD analysis and gain a more comprehensive understanding of the bonding characteristics, NCI analysis was performed, which can reveal subtle details about the types and strengths of interactions present in the system.^59−61^ It is important to note that while the NCI analysis provides valuable information about noncovalent interactions, it does not directly reflect the charge transfer processes observed in the CDD analysis. Instead, it offers complementary information, helping to build a more complete picture of the various forces in the adsorption systems. The combination of CDD and NCI analyses thus provides a more comprehensive understanding of the adsorption mechanism. The CDD analysis reveals the extent and spatial distribution of charge transfer, while the NCI analysis illuminates the landscape of weak, noncovalent interactions that also contribute to the overall binding process.

**Figure 4:**
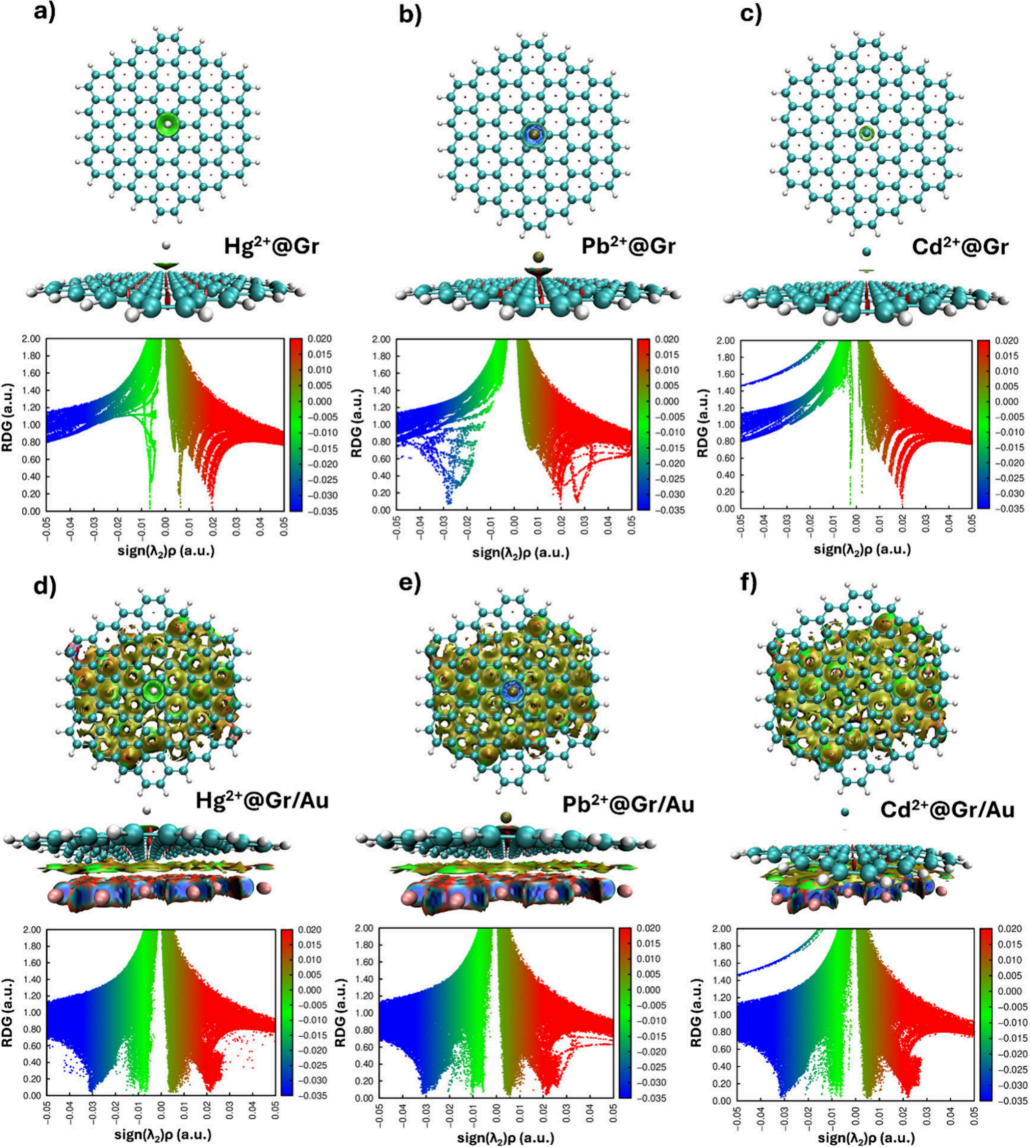
(Top and side view) plots of the noncovalent interaction (NCI) isosurfaces (reduced density gradient or RDG = 0.5) for HMIs adsorbed onto free-standing graphene (a, b, c) and Gr/Au heterointerface (d, e, f), respectively. The iso-surfaces are colored according to sign(λ_2_)ρ over the range −0.035 to 0.02 a.u. The NCI diagrams (RDG vs sign(λ_2_)ρ) describing the interaction between graphene, HMI, and substrate are depicted below. Red indicates steric repulsion region, green (light brown) indicates VdW interaction region, and blue implies the strong attractive interaction.

The results of the NCI analysis are shown in Figure 4. In the case of the Hg^2+^ on both free-standing graphene and Gr/Au(111), the color-filled map of the NCI iso-surface revealed a disc-like region in green and light brown colors (Figure 4a and Figure 4d). Correspondingly, the NCI diagram for Hg^2+^ on free-standing graphene (Note: the NCI diagram for mercury on Gr/Au(111) is significantly more complex due to its multifaceted nature, encompassing various types of interactions beyond just the cation-graphene surface interaction) shows the presence of two spikes in light green and light brown colors (points nearly approaching the bottom) in the negative and positive regions of sign(λ_2_)ρ. The red spike corresponds to steric interactions in the center of the hexagonal rings and is unrelated to the interaction between the Hg^2+^ and graphene. The interaction region marked by these colors can be identified as a van der Waals (VdW) interaction region. This observation aligns with the CDD analysis results, which showed significant charge accumulation around the Hg^2+^. The presence of VdW interactions, as indicated by the NCI analysis, suggests that the strong binding of Hg^2+^ to graphene involves both electrostatic and dispersion forces. This combination of interactions could explain the high adsorption energy and the extended charge accumulation region observed in the CDD analysis. Interestingly, in the case of Cd^2+^ adsorption (Figure 4c and Figure 4f), a similar pattern was observed, with the notable difference that the diameter of the disc-like region between Cd^2+^ and the graphene surface was significantly smaller than that between Hg^2+^ and graphene. The corresponding spikes were also shifted toward zero on the sign(λ_2_)ρ scale. This observation correlates well with the weaker charge transfer and smaller charge accumulation region seen in the CDD analysis for Cd^2+^, further supporting the notion of a weaker interaction between Cd^2+^ and graphene compared to Hg^2+^.

In contrast, Pb^2+^ adsorption on both surfaces led to a more complex interaction pattern (Figure 4b and Figure 4e). This is evidenced by the ellipsoid-like region between Pb^2+^ and graphene being colored in three distinct hues (red, blue, and green), with an emphasis on the blue-green (top view in both NCI iso-surfaces). NCI diagrams contained, in addition to the blue-green spike (indicating attractive interaction), an additional red spike shifted toward higher values of sign(λ_2_)ρ relative to the pre-existing red spike associated with steric effects inside the hexagonal rings. Essentially, the interaction between Pb^2+^ and graphene occurs through a balance between attractive interactions and steric repulsion. This complex interaction pattern for Pb^2+^ aligns with the CDD analysis results, which showed a distinct charge redistribution pattern, especially in the presence of a gold substrate. The balance between attractive and repulsive forces revealed by the NCI analysis could explain the intermediate binding strength of the Pb^2+^ ion compared to Hg^2+^ and Cd^2+^, as well as the unique charge redistribution observed in the CDD analysis. The NCI analysis thus provides a more nuanced understanding of the bonding characteristics, revealing that while all three ions interact with graphene primarily through noncovalent interactions, the nature and strength of these interactions vary significantly. Mercuric ion exhibits strong VdW interactions, which are consistent with its high binding energy and significant charge transfer. Cadmium shows weaker VdW interactions, aligned with its lower binding energy and minimal charge transfer. Conversely, lead presents a more complex interaction profile, balancing attractive and repulsive forces, which explains its intermediate binding strength and unique charge redistribution pattern.

### SPR Spectroscopy on the Gr/Au Heterostructures

**Figure 5:**
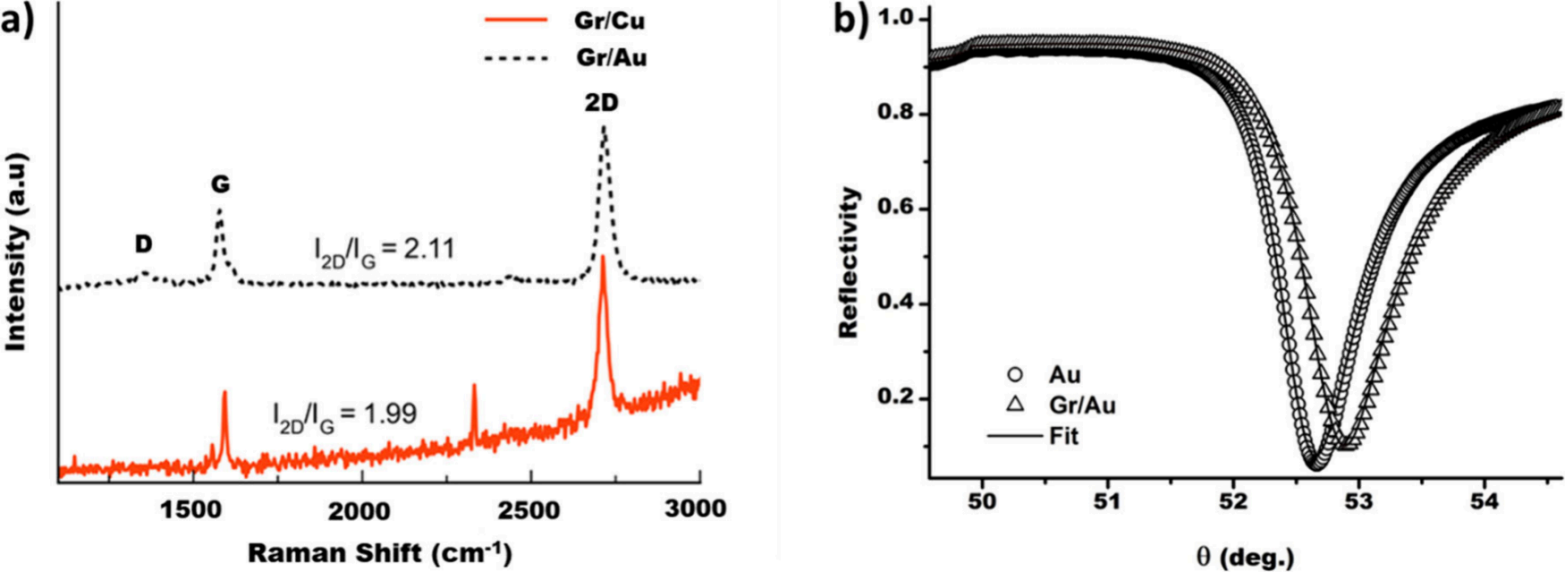
(a) Raman spectra on graphene grown on Cu (Gr/Cu) and graphene deposited on a gold thin film (Gr/Au). The I_2D_/I_G_ ratio of graphene on Au is 2.11, and 1.99 on Cu. The curved background seen in the Gr/Cu spectrum is due to the broad fluorescence of Cu oxide. (b) Experimental SPR spectra of the bare gold thin film (circles) and the Gr/Au heterostructure (triangles) in deionized water. The continuous lines represent the theoretical fit obtained using the values of thickness and dielectric constants reported in Table S1.

As reported in previous works,^9,16^ the quality and number of graphene layers grown on the Cu foil or deposited on the gold thin film can be verified by Raman spectroscopy. In Figure 5a are the reported Raman spectra associated with the Gr/Cu and Gr/Au structures. The spectra clearly indicate the presence of high-quality single-layer graphene on both Cu and Au substrates. Indeed, one may observe the expected D, G, and 2D bands characteristic of monolayer graphene in both substrates.^62^ The D band, located at around 1350 cm^–1^, is associated with disorder effects in the graphene layer. It arises from vibrations in the hexagonal carbon rings, adjacent to flake edges, impurities, and defects. The G band, located at around 1590 cm^–1^, corresponds to the in-plane rocking vibrations of the carbon atoms in the hexagonal ring. This vibrational mode is characteristic of sp^2^ carbon structures, and its position is sensitive to doping or strain in graphene layers. The 2D, located at about twice the frequency of the D band (about 1700 cm^–1^), arises from a two-phonon scattering process (second-order mode). The I_2D_/I_G_ intensity ratio is a valuable indicative to determine the number of graphene layers on a substrate.^63^ In our case, as shown in Figure 5, it can be seen that the I_2D_/I_G_ ratio is, in the case of the Au and Cu substrates, equal to 2.11 and 1.99, respectively, indicating that there is only one layer of graphene on top of the Cu and Au substrates. Furthermore, the full width half maximum (FWHM) of the 2D band of the Gr/Au sample is 34 cm^–1^, indicating that the transferred graphene is highly crystalline, despite the presence of disorder effects evidenced by the presence of the D band.

To characterize the optical properties of the Gr/Au heterostructures, it was used the same experimental approach reported in ref (9). Briefly, the SPR spectrum of two different regions of the samples was measured: a first region with bare gold and a second region where the graphene is deposited over the gold thin film (Gr/Au). In the latter case, it is known that the presence of water as an external medium creates a charge density difference (CDD) region extending from the first atomic layers of gold to the graphene and the first layers of water molecules, corresponding to an H_2_O/Gr/H_2_O/Au heterointerface with effective optical constants and an effective thickness that is, in general, different from the value of 0.3 nm associated with free-standing graphene.^9^ For the correct measurements of the effective parameters of the H_2_O/Gr/H_2_O/Au heterointerface, in this case, the SPR measurements were repeated using two different external media, deionized water (*n* = 1.33) and a glycerol water solution (*n* = 1.35). Then, using the same numerical procedure described in detail in ref (9), it was possible to determine the thickness and optical constants of the H_2_O/Gr/H_2_O/Au heterointerface, which were assumed to be the parameters of graphene in water.

In Figure 5b, the SPR spectra in deionized water of both the bare gold thin film and the Gr/Au heterostructure are shown, together with the theoretical fit obtained using the parameters reported in Table S1.

Using the SPR spectra of the Gr/Au heterostructures in deionized water and in glycerol water solution, it is straightforward to calculate the bulk sensitivity of these sensing platforms and to compare them with the sensitivity of the bare gold thin films. In these cases, the sensitivity to refractive index variation can be simply calculated as *S*_n_ = Δθ/Δ*n*, where Δ*n* = 0.02 represents the difference in the refractive index of the two external media (water and glycerol water solution), and Δθ is the angular shift after the change of the refractive index. It was obtained a sensitivity (*S*_n_) of 57°/RIU and 58°/RIU for bare gold thin film and Gr/Au heterostructures, respectively. This means that electron transfer in the H_2_O/Gr/H_2_O/Au heterointerface is responsible for an increase in the sensitivity of about 2% compared with bare gold thin films. This enhancement factor at the wavelength of 783 nm is in agreement with other reports in the literature.^9,64^

### Optical Sensing of HMIs by SPR Spectroscopy

**Figure 6:**
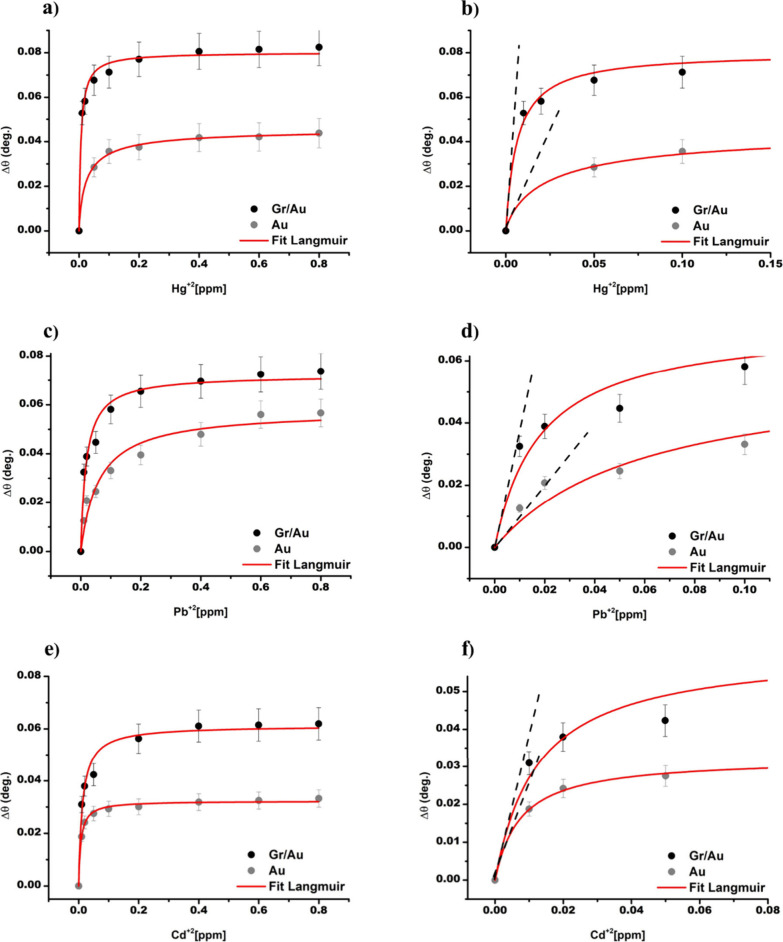
Shift of the resonance angle Δθ as a function of the concentration of HMIs in water using bare gold thin films (gray circles) or Gr/Au heterostructures (black circles) as SPR sensors. (a, b) Hg^2+^; (c, d) Pb^2+^; (e, f) Cd^2+^. The continuous red lines in all of the graphs represent the Langmuir fit on the data. The dashed black lines in parts b, d, and f are used to evaluate the slope of the first part of the curves, which is associated with the sensitivity of the sensors. The error bars in the graphs correspond to the standard deviation of Δθ calculated from the experimental reflectivity curve of three independent samples.

The shift in angle of resonance Δθ as a function of the concentration of the different HMIs for the bare gold thin film and Gr/Au heterostructures is shown in Figure 6. The corresponding SPR curves are shown in Figure S1 for both SPR sensing platforms. For each measurement, a Langmuir fit was performed on the SPR curve using eq 3, and the slope of the first part of the curve at low concentration was used to measure the sensitivity *S*_c_ and the LOD as defined by eq 2. The results of the Langmuir fit are shown in Table 2, together with the sensitivity *S*_c_ to the analyte concentration, the LOD, and the sensitivity enhancement *S*_*e*_ , defined as the ratio of the sensitivity of bare gold thin films and Gr/Au heterostructures, *S*_*e*_ = (*S*_c_^Gr/Au^/*S*_c_^Au^).

**Table 2:** Value of the Parameters of the Langmuir Fit (eq 3), Together with the Sensitivity to Analyte Concentration *S*_c_ and the LOD as Expressed by eq 1 and 2, Respectively; the Parameters Are Related to the Optical Sensing of the HMIs by Both Bare Gold Thin Films and Gr/Au Heterostructures; the Parameter*S*_*e*_ Represents the Sensitivity Enhancement after Graphene Transfer on the Gold Layer

SPR Chip	HMI	*S*_c_ (°/ppm)	*S*_*e*_	LOD (nmol L^–1^) (ppb)	Δθ_max_	K (L mol^–1^)	*R*^2^
Au	Hg^2+^	1.4	–	25	0.044	7.2 × 10^6^	0.996
				5.2			
	Pb^2+^	0.9	–	38	0.057	3.5 × 10^6^	0.955
				7.9			
	Cd^2+^	3.8	–	16	0.032	1.5 × 10^7^	0.994
				1.8			
Gr/Au	Hg^2+^	9.8	7.0	3.4	0.080	3.1 × 10^7^	0.980
				0.7			
	Pb^2+^	3.4	3.8	9.7	0.072	1.1 × 10^7^	0.966
				2.0			
	Cd^2+^	4.7	1.2	13	0.062	8.5 × 10^6^	0.970
				1.5			

While the change in refractive index sensitivity *S*_n_ after the graphene transfer to gold is about 2%, the increase in the sensitivity to concentration (within the analyte concentration ranges) were of 1.2 (20%) and 3.8 (280%) for Cd^2+^ and Pb^2+^ to a factor 7.0 (600%) for the Hg^2+^.

This result can be explained by considering the formation of a dynamic thin film of ions onto the graphene surface, which is responsible for a larger SPR angular shift than that associated with the variation in the refractive index of the external liquid. This is confirmed by the coefficient of determinations *R*^2^, indicating that the Langmuir isotherm model can be applied to describe the adsorption of HMIs on both bare gold surfaces^35^ and Gr/Au heterointerfaces. Particularly impressive is the performance of the Gr/Au sensors in the interaction with Hg^2+^ ions, associated with a maximum value of the thermodynamic affinity constant *K* = 3.1 × 10^7^ L mol^–1^, an enhancement of the sensitivity to concentration *S*_*e*_ ∼ 7, and the best limit of detection of about 3 nmol L^–1^ (0.7 ppb), below the limit established by the EPA.^30^

**Figure 7:**
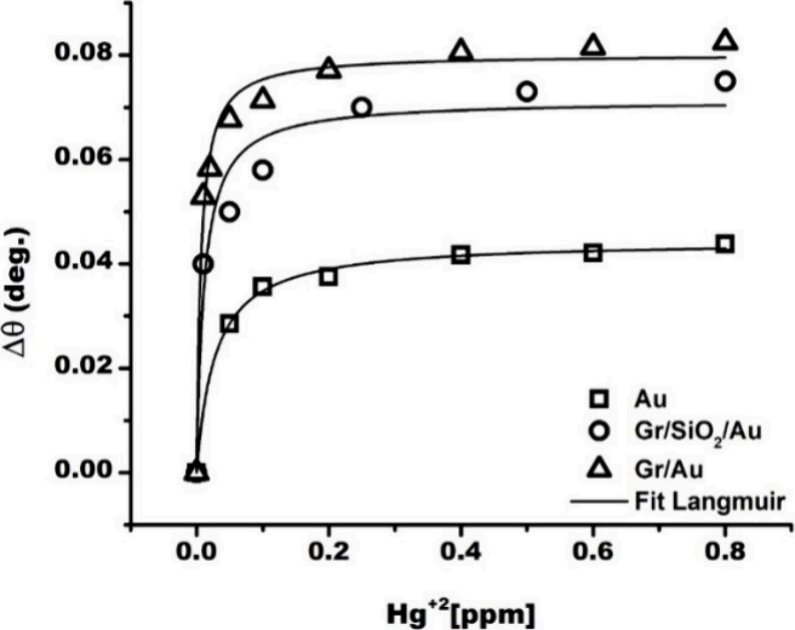
Shift of the resonance angle Δθ as a function of the concentration of the Hg^2+^ ions in water by the use of Au thin film, Gr/SiO_2_/Au, and Gr/Au heterostructures.

**Figure 8:**
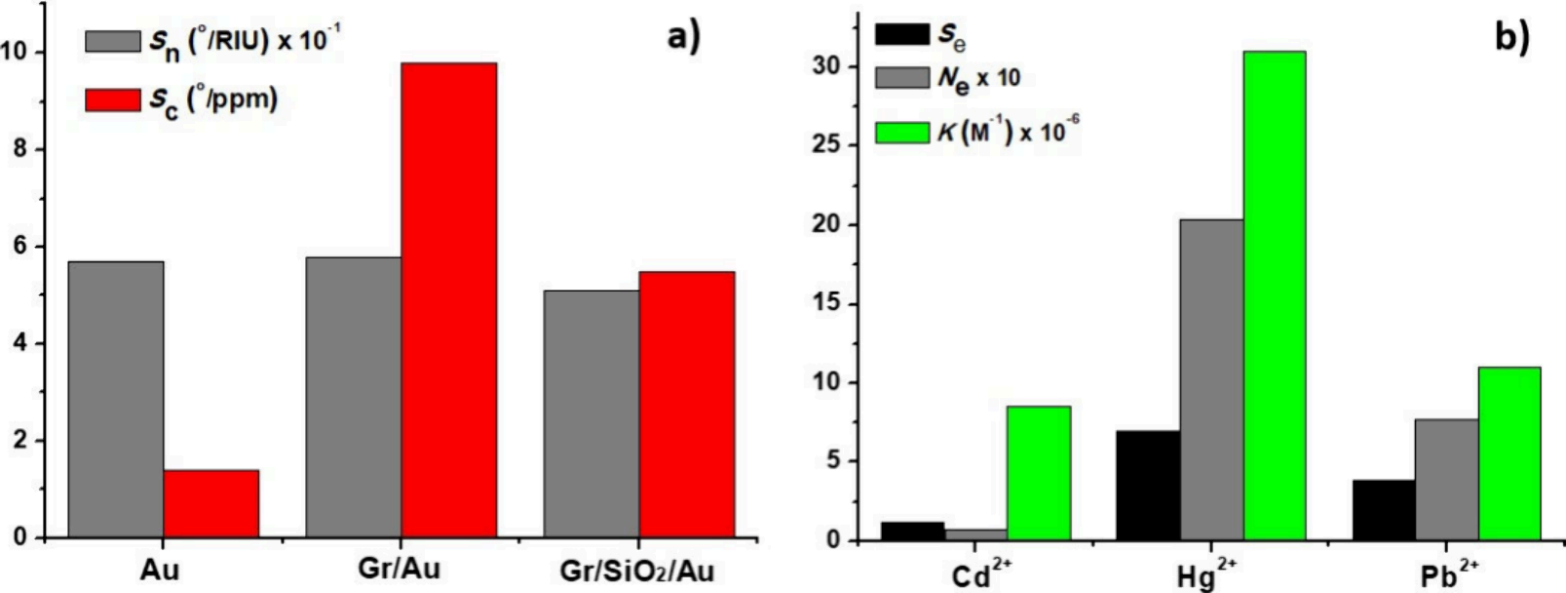
(a) Histogram showing the refractive index sensitivity *S*_n_ and the sensitivity with respect to the Hg^2+^ ions concentration *S*_c_ (eq 1). (b) Interaction of the Gr/Au heterostructure with different HMIs. Comparison of the experimental thermodynamic affinity constant *K*, the experimental values of the sensitivity enhancement *S*_e_, and the theoretical value of the number of electrons *N*_e_ transferred from the Gr/Au heterointerface to the HMIs. The latter was calculated from the Mulliken values given in Table 1.

To investigate the origin of the increase in response sensitivity to HMIs by using the Gr/Au heterostructures, the optical sensing of Hg^2+^ ions was repeated using three different structures: bare gold thin film, Gr/Au heterostructures, and graphene transferred onto a 50 nm SiO_2_ thin film deposited over the gold layer (Gr/SiO_2_/Au). The latter configuration is used to experimentally simulate the affinity of the HMI to graphene when there is no interaction with the gold layer. The experimental curves of the resonance angle shift are shown in Figure 7, while Figure 8a presents the comparison between the refractive index sensitivity *S*_n_ and the sensitivity *S*_c_ expressed in terms of the Hg^2+^ concentration for the three different structures of the SPR chip. The values of both *S*_n_ and *S*_c_, together with the affinity constant *K* are reported in Table 3.

**Table 3:** Value of the Parameters of the Langmuir Fit (eq 3), Together with the Sensitivity to Analyte Concentration*S*_c_ and the LOD as Expressed by eq 1 and 2, Respectively; the Parameters Are Related to the Optical Sensing of the Hg^2+^ Ion by Bare Gold Thin Films, Gr/Au, and Gr/SiO_2_/Au Heterostructures

Interface	HMI	*S*_c_(°/ppm)	LOD (ppm)	Δθ_max_	K (L mol^–1^)	*R*^2^
Au	Hg^2+^	1.4	5.2 × 10^–3^	0.044	7.2× 10^6^	0.996
Gr/SiO_2_/Au	Hg^2+^	5.5	1.2 × 10^–3^	0.071	1.8 × 10^7^	0.947
Gr/Au	Hg^2+^	9.8	0.7 × 10^–3^	0.079	3.1 × 10^7^	0.975

The *S*_c_ value of 5.5 *°*/ppm associated with the interaction of the Hg^2+^ with Gr/SiO_2_/Au heterostructures is much lower than that of the Gr/Au heterostructure, showing that the binding affinity of the ions depends on the substrate on which the graphene is transferred and is higher when the graphene is in direct contact with the gold.

Figure 8b shows the comparison between the experimental performances of the Gr/Au heterostructures in the SPR sensing of the different HMIs and the theoretical calculations on the electrons *N*_e_ transferred to the HMIs. With respect to the maximum sensitivity to Hg^2+^ ions, it can be observed that the Hg^2+^@Gr/Au heterointerfaces are characterized by the highest electron transfer to the HMI, which is translated into a net charge that is even less negative in the Mulliken approach (Table 1). According to the atomic dipole moment corrected Hirshfeld (ADCH) charge analysis, the Au layer loses the electrons, initially donated by graphene, after the adsorption of Hg^2+^.

From Figure 3f and Figure 8b, it is noticed that no significant CDD is observed for the Cd^2+^@Gr/Au heterostructure, with a maximum absolute value of the surface integrated CDD of about 0.02. This contrasts with the Hg^2+^@Gr/Au heterointerface (see Figure 3b), characterized by a maximum absolute value of the surface integrated CDD of about 0.45, in a region extending to about 1 nm.

It is shown in Figure 8b that both the sensitivity enhancement *S*_e_ and the thermodynamic affinity constant *K* are correlated with the number of electrons transferred to the HMIs. These results suggest that two features of the HMI-surface interaction have a significant influence on the SPR response of the optical sensors: (i) the sign of the binding energy, which promotes the formation of a thin film by spontaneous adsorption on the binding sites when it is negative, and (ii) the number of electrons *N*_e_ transferred to the HMIs, with the extension of the CDD region along the HMI@Gr/Au heterointerface. As reported in ref (9), the characteristics of the CDD actually control the effective thickness and refractive index of the heterointerface formed after the interaction with the analytes, and ultimately the sensitivity *S*_c_ of the SPR chips.

**Figure 9:**
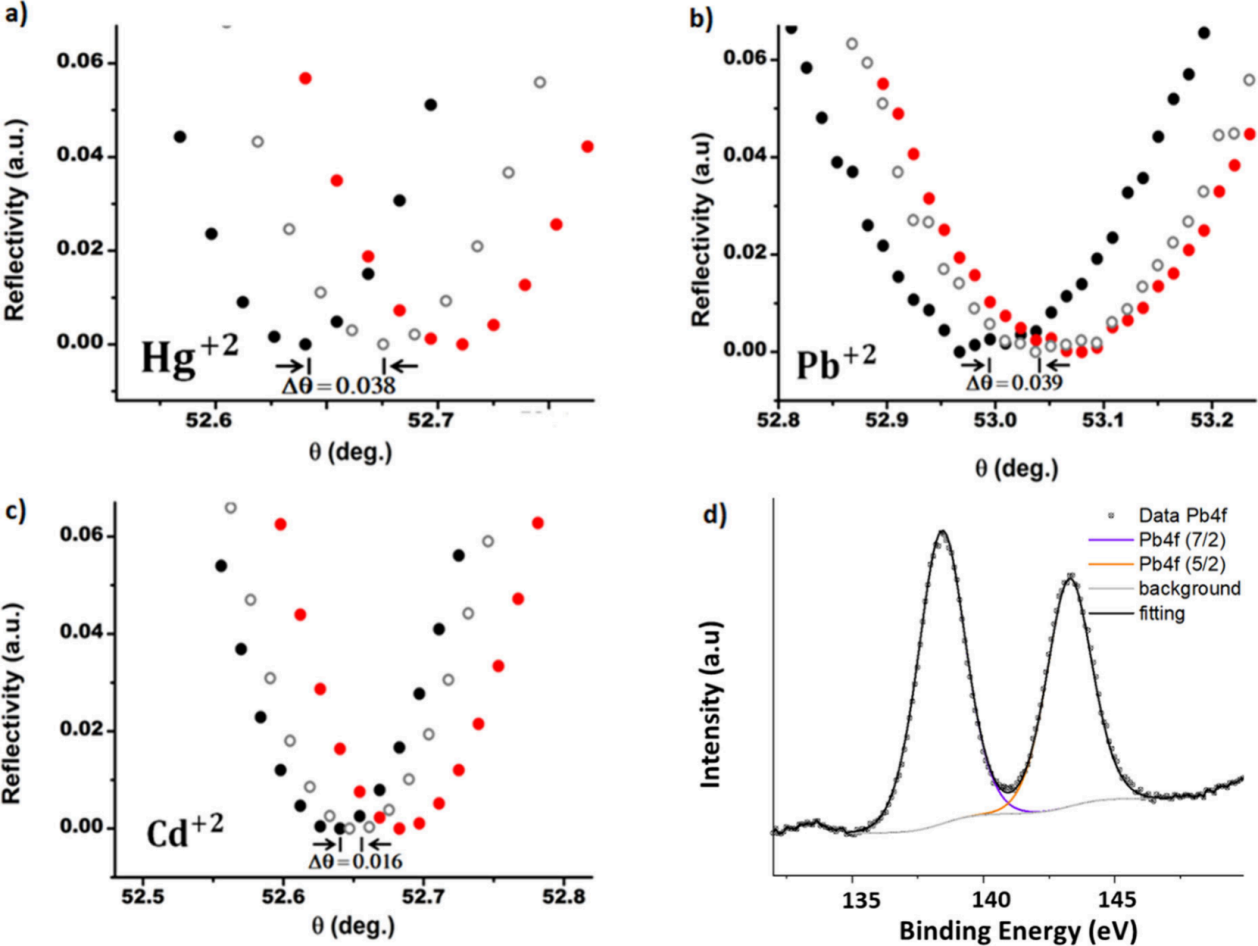
(a–c) Permanent shift of the SPR resonance angle after the interaction of the Gr/Au heterointerfaces with the HMI ions. Black circles: before HMI injection. Red circles: after injection of HMI at 1 ppm. White circles: after rinsing with deionized water. (d) XPS measurements on the Gr/Au heterointerfaces after interaction with the Pb^2+^ ions at 1 ppm after rinsing with deionized water.

In order to study the nature of the film of HMIs formed on the SPR substrates, deionized water was gently flowed to rinse the surface of the sensors after the interaction with the analytes at a concentration of 1 ppm. As shown in Figure 9a–c, it was observed that the SPR spectra were characterized by a permanent angular shift Δθ for all the HMIs with values between 0.016° and 0.088°, which is associated with the formation of a stable thin film of HMIs. Nevertheless, an attractive interaction between the HMIs and the Gr/Au heterointerfaces is indeed expected from the plots of the noncovalent interaction (NCI) iso-surfaces reported in Figure 4.

To verify the presence of the HMIs on the surface of the Gr/Au heterointerfaces, XPS analysis was performed on the samples after fluxing 1 ppm of the HMIs followed by gentle rinsing with deionized water. The spectrum of the Pb 4f region is shown in Figure 9d. The position of the Pb 4f_7/2_ peak is shifted by about 1.5 eV from the expected position of the Pb metallic (the position of the Pb peak in the spectrum shown in Figure 9d is ∼138.3 eV), indicating the presence of Pb^2+^ species adsorbed on the Gr/Au heterointerfaces. Since XPS did not detect nitrogen atoms in the survey spectra (Figure S2), it was considered that the peak shifts with charge transfer in the Pb^2+^@Gr/Au heterostructures (Table 1). Additionally, the Au 4f peak showed a redshift of 0.4 eV when compared with the metallic state (Au^0^), as depicted in Figure S2b. That could also indicate electron transfer to the Pb cations in this type of interface.

The selective XPS detection of Pb compared to the other metals, can be explained considering the high volatility of reduced mercury in comparison to lead^65^ and the theoretical results on the binding energy of the HMI on the heterostructures (Table 1). The latter show that the Cd^2+^@Gr/Au heterointerface is characterized by the lowest (in absolute value) values of binding energy and electron transfer to the HMI, which translates into the lowest value of thermodynamic binding affinity (Figure 8) and a smaller amount of Cd species permanently interacting with the Gr/Au, ultimately undetectable by XPS spectroscopy.

To conclude the discussion on the experimental results, it is interesting to compare the presented results with the performance reported in the literature on the use of graphene oxide (GO), reduced graphene oxide (rGO), or graphene-metal oxide heterointerfaces (Gr/MO-hetero) for the detection of HMIs. For this reason, the main characteristics of the sensor performance, such as detection method, selectivity, and LOD versus analyte concentration, are listed in Table 4. For a complete comparison with the achieved results, the performance of traditional SPR sensors for HMIs based on organic layer/gold heterointerfaces was also reported.

**Table 4:** Performance of Different Heterostructures for the Detection of HMIs in Water: Sensor Structure, Limit of Detection (LOD), and Selectivity

HMI	Sensor Structure	Detection Method	LOD (nmol L^–1^)	Selectivity	Reference
Hg^2+^	**Au**	**SPR**	25	**NO**	**This work**
	**Gr/Au**	**SPR**	3.4	**NO**	**This work**
	PPy-CHI/Au^a^	SPR	2.5 × 10^3^	NO	(66)
	GO/Au	SWV^i^	4.0	NO	(67)
	1,6-hexanedithiol/Au	SPR	1.0	YES	(68)
	rGO/AuNPs	FET	1.0	YES	(26)
	Gr/SPE^b^	SWASV^j^	1.5 × 10^2^	YES	(69)
	TGA@rGO/Au^c^	FET		YES	(25)
Pb^2+^	**Au**	**SPR**	**45**	**NO**	**This work**
	**Gr/Au**	**SPR**	**8.7**	**NO**	**This work**
	PPy-CHI/Au	SPR	2.5 × 10^3^	NO	(66)
	rGO/SnO	SWASV	0.18	NO	(34)
	BCAT-CHI/Au^d^	SPR	1.5 × 10^2^	YES	(70)
	GSH@rGO/AuNPs^e^	FET	10	YES	(71)
	Glycine-rGO-Polyaniline	CV^k^	7 × 10^–2^	YES	(72)
	l-Cysteine PEDOT:PSS/rGO^f^	CV	0.5	YES	(73)
Cd^2+^	**Au**	**SPR**	**16**	**NO**	**This work**
	**Gr/Au**	**SPR**	**13**	**NO**	**This work**
	MT-Dextran/Au^g^	SPR	10	NO	(74)
	rGO/SnO	SWASV	0.1	NO	(34)
	U-SPDP/Au^h^	SPR	2 × 10^4^	YES	(75)
	Glycine-rGO-Polyaniline	CV	7 × 10^–2^	YES	(72)
	Thiacalix[4]arene/Gr	FET	1 × 10^3^	YES	(76)
	Bi_2_O_3_–Fe_2_O_3_-GO	SWV	1.7 × 10^–2^	YES	(77)

a Polypyrrole-chitosan (PPY-CHI).

b Screen sprint electrode (SPE).

c Thioglycolic acid (TGA).

d p-*tert*-Butylcalix[4]arene-tetrakis immobilized in chitosan thin film (BCAT-CHI).

e l-Glutathione (GSH).

f Poly(3,4-ethylenedioxythiophene):poly(styrenesulfonate) (PEDOT:PSS).

g Metallothionein on a carboxymethylated dextran matrix (MT-Dextran).

h Urease modified N-succinimidyl 3-(2-pyridyldithiol) propionate (U-SPDP).

i Square wave voltammetry (SWV).

j Square wave anodic stripping voltammetry (SWASV).

k Cyclic voltammetry (CV).

SPR sensors for HMIs are based on the recognition of the ions by organic thin films over the gold layers,^66,70,75^ some of which give rise to a selective interaction with specific HMIs.^70,75^ In terms of LOD, the Gr/Au heterostructures presented in this research are orders of magnitude better than PPy-CHI/Au or U-SPDP/Au devices,^66,75^ and comparable to MT-Dextran/Au and 1,6-hexanedithiol/Au structures,^68,74^ although the latter have the advantage of a selective recognition for Hg^2+^.

GO/Au, rGO/Au, or rGO/AuNPs structures are also used in electronic sensors^25,26,67,71^ based on field effect transistors (FET) or square wave voltammetry (SWV), and are characterized by LOD values similar to the proposed devices. The addition of organic ligands to act as specific receptor molecules, such as thioglycolic acid (TGA) or reduced l-glutathione (GSH), can also be used in these devices to achieve specificity toward selected ions.^25,71^

The presented scenario indicates that the SPR devices based on Gr/Au heterointerfaces are characterized by an LOD value comparable to that of the most sensitive sensors based on optical or electrical measurements. Moreover, the results obtained on the affinity constant of Hg^2+^@Gr/Au, which is about four times higher than that of the other HMI@Gr/Au heterostructures without the use of specific receptor molecules, suggest the possibility to investigate in the near future the application of interference buffering strategies to obtain a specificity toward Hg^2+^ ions.

## Conclusions

The interaction between divalent HMIs (Hg^2+^, Pb^2+^, Cd^2+^) and graphene-gold heterointerfaces has been studied through theoretical DFT calculations and experimental SPR spectroscopy in aqueous environment. The theoretical results indicate a negative binding energy between all the HMIs and the heterostructures and a charge transfer from the Gr/Au structure to the HMI. While the charge transfer to the Cd^2+^ is negligible, the Hg^2+^ is completely reduced, with a maximum absolute value of the surface integrated charge density difference of about 0.45 in a region extending to about 1 nm. Using SPR spectroscopy, it was experimentally verified that a Langmuir isotherm model can well describe the adsorption of the HMIs on the heterointerfaces supporting the surface plasmon polariton. A thermodynamic affinity constant as high as *K* = 3.1 × 10^7^ L mol^–1^ is observed for Hg^2+^@Gr/Au heterostructures, compared to 1.1 × 10^7^ L mol^–1^ and 8.5 × 10^6^ L mol^–1^ for Pb^2+^@Gr/Au and Cd^2+^@Gr/Au, respectively. Compared to the bare gold surface, the graphene-gold devices are characterized by an enhancement of the sensitivity to analyte concentration, which, interestingly, correlates with the theoretical number of electrons transferred to the HMIs. While a small enhancement is observed for Cd^2+^ ions, an enhancement of almost an order of magnitude is observed for Hg^2+^ ions, for which we obtain an impressive sensitivity of about 0.01°/ppb and a limit of detection of 0.7 ppb (∼3 nmol L^–1^), below the EPA recommendation for drinking water.

## Supporting Information Available

SPR characterization of the Gr/Au heterostructures; HMIs optical sensing; XPS measurements (PDF)
