# Supplementary material for: On the Strong Binding Affinity of Gold-Graphene Heterostructures with Heavy Metal Ions in Water: A Theoretical and Experimental Investigation
Source: Langmuir. 2024 Sep 13;40(38):20204–18. doi: 10.1021/acs.langmuir.4c02568 (PMC11448048; doi:10.1021/acs.langmuir.4c02568)
Supplement: Supplementary file 1 — la4c02568_si_001.pdf [file la4c02568_si_001.pdf]

Supporting Information

On the strong binding affinity of gold-graphene heterostructures with heavy metal ions in water: a theoretical and experimental investigation

Tommaso Del Rosso[1,*], Ivan Shtepliuk[2,*], Quaid Zaman[1,3], Luis Gonzalo Baldeón Huanqui[1], Tahir[1], Fernando Lazaro Freire[1], Andre Nascimento Barbosa[1], Marcelo Eduardo Huguenin Maia da Costa[1], Ricardo Q. Aucelio[4], Jarol Ramon Miranda Andrades[4], Cesar D. Mendoza[1,5], Rajwali Khan[6], Giancarlo Margheri[7]

[1]Department of Physics, Pontifícia Universidade Católica do Rio de Janeiro, Rua Marques de São Vicente, 22451- 900, Rio de Janeiro, Brazil
[2]Semiconductor Materials Division, Department of Physics, Chemistry and Biology - IFM, Linköping University, S-58183 Linköping, Sweden
[3]University of Buner, Department of Physics, Main Sowari Bazzar, 17290 Buner, Pakistan
[4]Department of Chemistry, Pontifícia Universidade Católica do Rio de Janeiro, Rua Marques de São Vicente, 22451- 900, Rio de Janeiro, Brazil
[5]Departamento de Engenharia Elétrica, Universidade do Estado do Rio de Janeiro, UERJ, Rua São Francisco Xavier 524, Maracanã, Rio de Janeiro, 20550-900, RJ, Brazil
[6]National Water and Energy Center, United Arab Emirates University, P.O Box 17551, Sheik Khalifa Bin Zayed Street 1, Al-Ain, United Arab Emirates
[7]Istituto dei Sistemi Complessi Sezione di Sesto Fiorentino (I.S.C - CNR), Via Madonna del Piano 10, 50019 Sesto Fiorentino, Italy

Corresponding authors: tommaso@puc-rio.br; ivan.shtepliuk@liu.se

Contents

Cover page …………………………………………………………………………………………..S1

SPR characterization of the Gr/Au heterostructures……………………………………………..S2

Table S1: Thickness and dielectric constant (ε_1 + i ε_2) of the different layers constituting the SPR heterostructure……S2

HMIs optical sensing…………………………………………………………………………....S2-S3

Fig.S1: Shift of the SPR curves in function of the concentration of HMIs in water using bare gold thin film and Gr/Au heterostructures as SPR sensors. (a,b) Hg^{2+}; (c,d) Pb^{2+}; (e,f) Cd^{2+}. (a,c,e) Bare gold thin films. (b,d,f) Gr/Au heterostructures. ………………………………………………………………………………………......S3

XPS measurements………………………………………………………………………………… S4

Fig.S2: a) Survey spectra of the XPS measurements on the Gr/Au heterointerfaces after interaction with the Pb^{2+} ions. b) Au4f peak of the Gr/Au heterointerfaces after interaction with the Pb^{2+} ions. Au4f peak (7/2) position was 83.6eV, showing a redshift of 0.4eV when compared with metallic Au. ……………………………………………………………………S4

SPR characterization of the Gr/Au heterostructures

In Table S1 are reported the thickness and the real and imaginary part of the dielectric constant of the layers constituting the SPR heterostructure. Herein, as graphene parameters (Gr), are reported the thickness and optical constant of the H_2O/Gr/H_2O/Au heterointerface, which is produced by the interaction between the first atomic layers of gold, the graphene and the first layers of water molecules.

Layer	t(nm)	ε_1	ε_2
SF4	0	3.022	0
Au	52.33	-24.67	1.69
Gr	1.06	6.76	2.84
Water	0	1.77	0

Table S1: Thickness and dielectric constant (ε_1 + i ε_2) of the different layers constituting the SPR heterostructure.

HMIs optical sensing

In Figure S1 we show the shift of the SPR curves of bare gold sensors and Gr/Au heterostructures after the interaction with the different HMIs. These SPR spectra were used to build the graphs reported in Figures 6 and 7 of the main text.

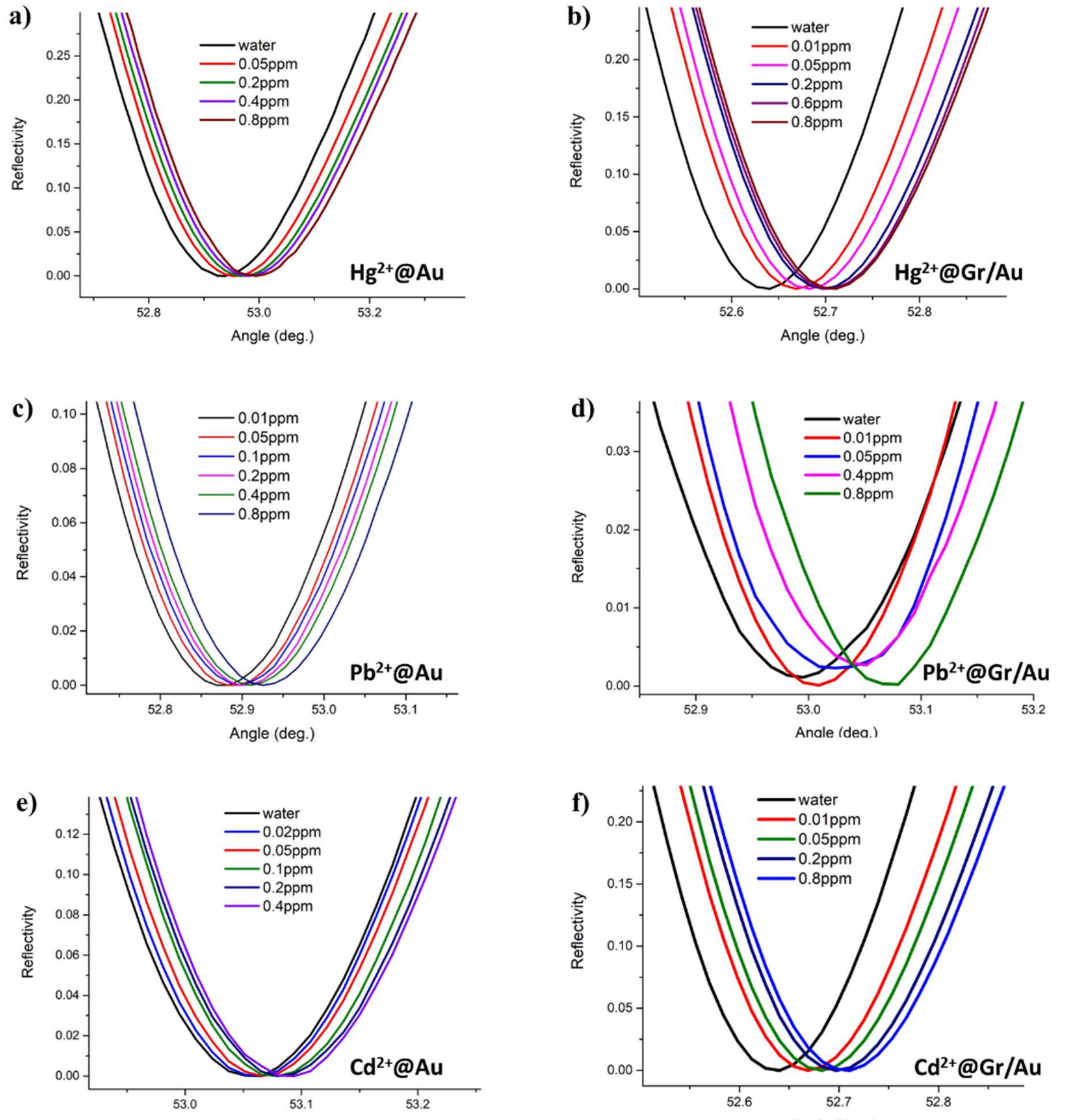

Fig.S1: Shift of the SPR curves in function of the concentration of HMIs in water using bare gold thin film and Gr/Au heterostructures as SPR sensors. (a,b) Hg^{2+}; (c,d) Pb^{2+}; (e,f) Cd^{2+}. (a,c,e) Bare gold thin films. (b,d,f) Gr/Au heterostructures.

XPS measurements

In Figure S2 it is represented the survey of the XPS spectra of the Pb^{2+}@Gr/Au heterostructures. The signal of gold is lower than carbon due the shielding effect of the graphene over the noble metal. The presence of oxygen is probably due to surface contamination or partial oxidation after exposition to the atmosphere. The position of the $Pb4f_{7/2}$ peak is shifted from the metallic position by around 1.5eV (observed position of Pb peak in the spectrum at ~138.3 eV). Figure S2b represents the Au4f peak of the Gr/Au heterointerfaces after interaction with the Pb^{2+} ions. Au4f peak (7/2) position was 83.6eV, showing a redshift of 0.4eV when compared with metallic Au.

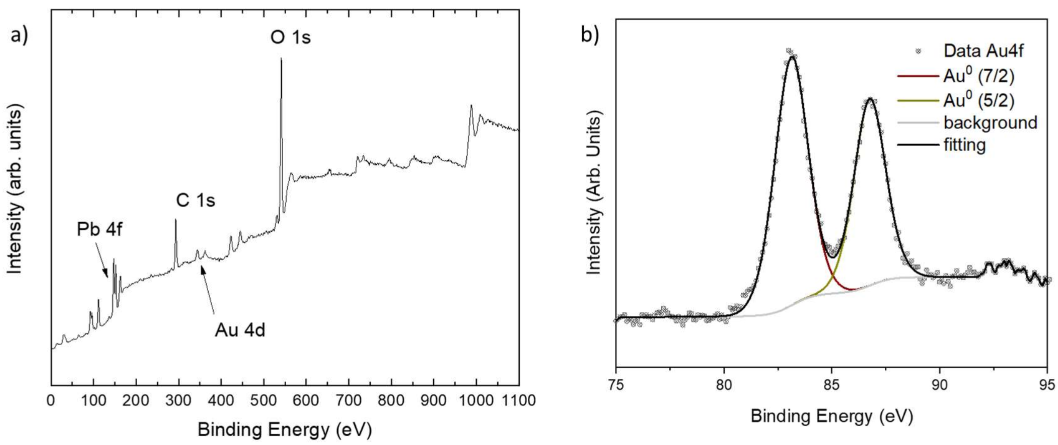

Fig.S2: a) Survey spectra of the XPS measurements on the Gr/Au heterointerfaces after interaction with the Pb^{2+} ions. b) Au4f peak of the Gr/Au heterointerfaces after interaction with the Pb^{2+} ions. Au4f peak (7/2) position was 83.6eV, showing a redshift of 0.4eV when compared with metallic Au.
